# Ischemic stroke-specific cognitive dysfunction: a bibliometric analysis of research evolution

**DOI:** 10.3389/fneur.2026.1891094

**Published:** 2026-09-04

**Authors:** Wenliang Ma, Lan Luo, Bingcang Huang, Ting Liu, Ying Wang

**Affiliations:** 1 School of Gongli Hospital Medical Technology, University of Shanghai for Science and Technology, Shanghai, China; 2 Department of Radiology, Pudong Gongli Hospital, Shanghai University of Medicine & Health Sciences, Shanghai, China; 3 Department of Central Laboratory, Pudong Gongli Hospital, Shanghai University of Medicine & Health Sciences, Shanghai, China; 4 Traditional Chinese Medicine Research and Development Laboratory, Pudong Gongli Hospital, Shanghai University of Medicine & Health Sciences, Shanghai, China

**Keywords:** bibliometrics, cognitive dysfunction, inflammation, ischemic stroke, prediction models

## Abstract

**Background:**

Post-Stroke Cognitive Impairment (PSCI) is a common complication after stroke, significantly affecting patients’ quality of life and long-term prognosis. Although PSCI has been extensively studied, most research combines ischemic and hemorrhagic strokes. Whether the distinct inflammatory mechanisms and vascular injury underlying ischemic stroke-specific cognitive dysfunction (ISCD) have followed an independent research trajectory remains unknown from a bibliometric perspective.

**Methods:**

Using the Web of Science Core Collection (2004–2024), our study identified 1,062 articles focused on ISCD for bibliometric analyses. Following cross-validation with PubMed confirming complete literature concordance, bibliometric tools (CiteSpace, VOSviewer, the R-based bibliometrix package) were employed to analyze publication trends, contributions and collaborations among countries, institutions, journals, and authors. Subgroup analyses quantified trends in inflammatory biomarkers, assessment tools and prediction models.

**Results:**

Annual publication peaked in 2022 (*n* = 133), followed by a decline in 2023 and 2024. China dominated in productivity (31.4%), while the United States exhibited the highest collaborative centrality (0.30). Keyword bursts identified ischemic-specific hotspots, including “model” (2021, burst strength = 4.66), “validation” (2022, burst strength = 4.96), and “inflammation” (2021, burst strength = 3.83). Inflammatory biomarkers and assessment tools have consistently been research hotspots in ISCD, recent efforts are shifting toward the development of prediction models for cognitive outcomes following acute ischemic stroke.

**Conclusion:**

This ischemic-specific bibliometric analysis confirms that inflammatory biomarkers, assessment tools, and prediction models are persistent hotspots in ISCD, with a clear ongoing shift from descriptive studies toward predictive model development and validation. These findings provide a quantitative literature-based rationale for guiding future research directions.

## 1 Introduction

Stroke represents the second leading cause of global mortality, with ischemic stroke (IS) constituting its predominant subtype (approximately 80% of cases) (1). This cerebrovascular pathology is characterized by cerebral artery occlusion, resulting in ischemia, hypoxia, and consequent structural and functional neurological damage, with post-stroke cognitive impairment (PSCI) affecting nearly two-thirds (67%) of survivors, significantly impacting functional recovery and long-term quality of life (2, 3).

In IS, tissue hypoxia and metabolic disorders lead to cell damage or death, releasing damage-associated molecular patterns (DAMPs) such as high-mobility group box 1 (HMGB1), ATP, and heat shock proteins (4). These molecules are recognized by immune cells after reperfusion, thereby inducing the release of proinflammatory cytokines (e.g., IL-6, TNF-α), while activating the complement system and neutrophil infiltration, exacerbating inflammatory damage (5). The inflammatory response, in turn, exacerbates reperfusion injury through mechanisms such as immune activation and vascular damage (6). This interaction forms a vicious cycle that constitutes a core pathological mechanism of ischemic stroke, seriously affecting the prognosis of patients’ neurological function and cognitive outcomes.

Given the distinct pathophysiological mechanisms underlying ischemic stroke-specific cognitive dysfunction (ISCD) and its substantial clinical prevalence, a systematic analysis of the research landscape is urgently needed. Bibliometric analysis has emerged as a powerful tool to quantitatively assess the development, collaboration, and emerging trends within scientific fields (7). This methodology has been increasingly applied across various medical disciplines, including stroke research, to map knowledge structures, identify key contributors, and forecast frontier topics (8, 9). For instance, recent bibliometric studies have illuminated research evolution in areas such as stem cells in stroke treatment (10), inflammation and mitochondrial stress in stroke (11). However, despite the proliferation of bibliometric reviews in neurology, no comprehensive analysis has specifically focused on the thematic evolution and research priorities of ISCD using both bibliometric and visual analytic approaches. Therefore, this bibliometric study aims to systematically delineate the research landscape of ISCD, with a particular focus on identifying key research hotspots and emerging trends. Ultimately, we seek to deepen the understanding of this disease and provide guidance for future innovation.

## 2 Materials and methods

### 2.1 Data collection and search strategy

To ensure the comprehensiveness of the literature data and examine potential selection bias associated with using a single database, our study performed a cross-validation analysis by supplementing the Web of Science Core Collection (WoSCC) with the PubMed database. Our search terms were derived from the Medical Subject Headings (MeSH) framework for “Ischemic Stroke” and “Cognitive Dysfunction” in PubMed. For WoSCC, we used equivalent keyword combinations to ensure consistent coverage. The complete search strategy and results are provided in Supplementary Tables 1, 2. The literature search was conducted on February 7, 2025. The search period was 2004–2024 for both databases. A comparison of the retrieval results revealed that, given our specific search terms and time frame, all core publications were indexed in WoSCC, and no unique publications were identified exclusively in PubMed. This complete overlap can be explained by the fact that WoSCC, as the “gold standard” for bibliometric analyses, applies a rigorous journal selection and curation process (12), whereas high-quality literature in established research fields is typically indexed in both databases, even though PubMed has a broader but less stringent indexing policy. Given the superior metadata standardization of WoSCC for bibliometric tools, we selected WoSCC as the sole data source for subsequent analyses.

**Figure 1:**
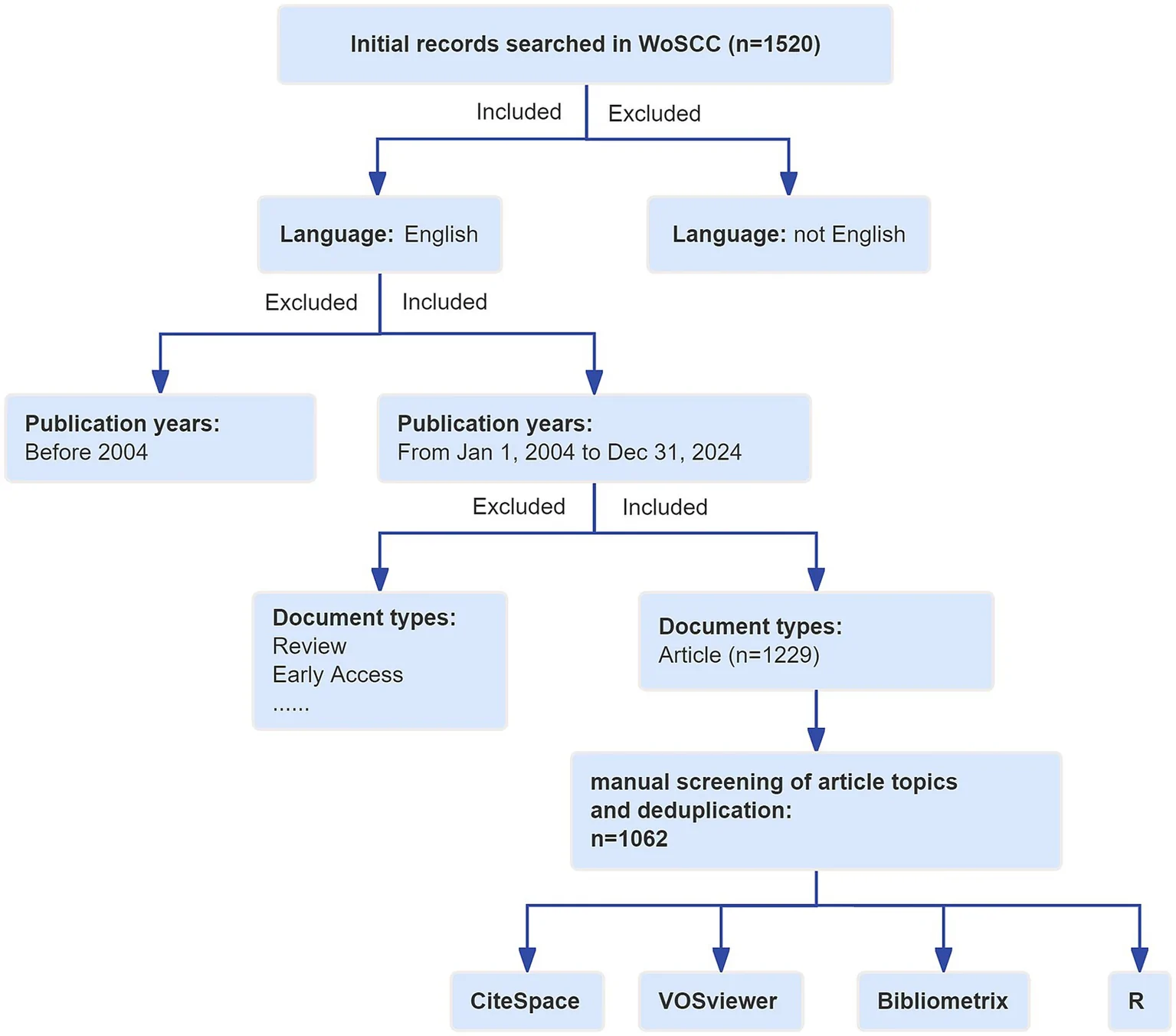
Literature search and screening process of this study.

To capture evolving research trends while maintaining focus on primary scientific contributions, we limited our search to document type “Article,” published between 2004 and 2024. The language was restricted to English. We then manually screened the titles and abstracts of the retrieved records against predefined inclusion and exclusion criteria. The inclusion criteria were: (i) studies explicitly addressing ISCD as a primary focus, including but not limited to investigations into its pathogenesis, neuroimaging features, risk factors, biomarkers, assessment methods, interventions, or rehabilitation outcomes; and (ii) studies involving patients with IS, or basic experimental research utilizing well-established and broadly recognized animal models of IS. Studies were excluded if they: (i) exclusively focused on general cognitive aging, Alzheimer’s disease, or other secondary cognitive impairments unrelated to IS, without clearly identifying IS as the core exposure or etiological factor; and (ii) lacked direct relevance to ISCD, such as those solely evaluating the efficacy of acute-phase thrombolytic therapy or endovascular thrombectomy without addressing cognitive outcomes. Finally, a total of 1,062 unique articles were included for subsequent analysis. Figure 1 illustrates the entire screening process.

### 2.2 Data analysis

The exported data were imported into CiteSpace (version 6.4.R1) for an initial removal of duplicate records, thereby ensuring a non-redundant dataset (13). Subsequent analyses identified key metrics, including publication volume, authorship, institutional and national contributions, journal distribution, keyword trends, and citation patterns. In the “Selection Criteria,” the “g-index” was uniformly set to K = 5 to encompass all nodes, with default parameters of TOP *N* = 50 and TOP *N*% = 10.0%.

The dataset was also analyzed using the bibliometrix package in R (version 4.4.1) and VOSviewer (version 1.6.20) to generate quantitative bibliometric indicators and visualizations (14). VOSviewer was primarily employed for constructing and visualizing collaboration networks and keyword co-occurrence maps, focusing on the strength of associations between items. The R package Bibliometrix was used for descriptive statistical analyses and trend plotting.

To comprehensively characterize the ISCD research landscape, we first analyzed publication and citation volume to gain an overview of productivity and development trends. We then examined country contributions and international collaboration to delineate the global cooperative network. Institutional performance was assessed to recognize major research centers and their inter-organizational partnerships. Subsequently, we conducted journal and co-cited journal analyses to outline the core publication outlets and disciplinary knowledge base. We evaluated authors and co-cited authors to pinpoint key opinion leaders and influential research groups. Furthermore, keyword co-occurrence analysis was performed to uncover research hotspots and thematic clusters. Finally, we employed reference analysis to trace the intellectual origins and knowledge evolution of the field. These analyses together formed the basis for our systematic bibliometric analysis.

## 3 Results

### 3.1 Analysis of publication and citation trends

**Figure 2:**
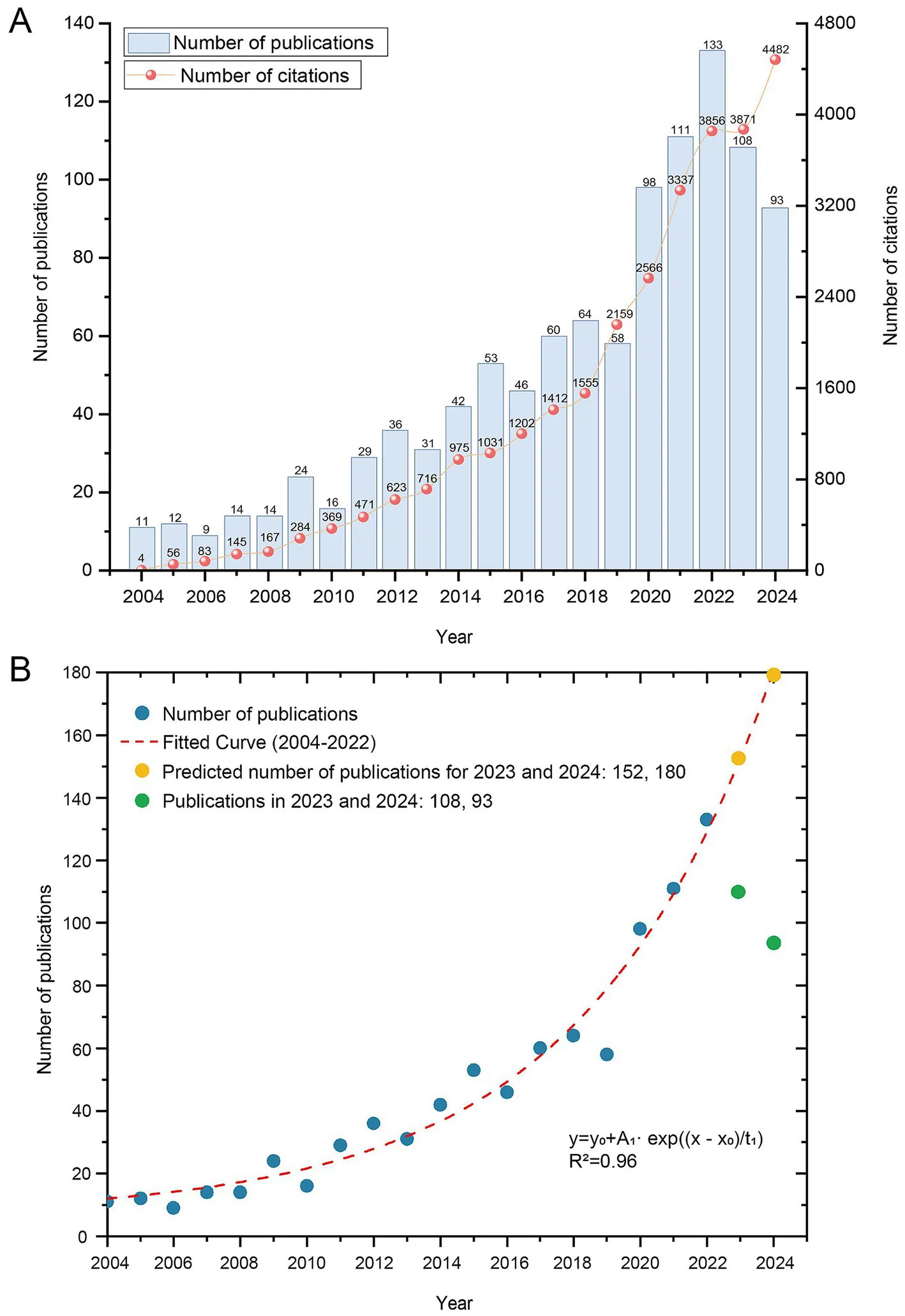
Trends of annual publications and citations in ISCD field from 2004 to 2024. **(A)** Annual number of publications and citations from 2004 to 2024. **(B)** The fitted curve of annual publications and the contrast between predicted and actual publications.

Figure 2 illustrates the temporal evolution of ISCD-related publications and citations from 2004 to 2024. The publication count evolved through different phases (Figure 2A). The period from 2004 to 2019 was marked by steady though fluctuating growth, followed by a sharp rise between 2019 and 2020. Annual publications exceeded 100 in 2021 (*n* = 111), culminating in a peak of 133 publications in 2022. However, subsequent years (2023–2024) witnessed a notable decline in output. As shown in Figure 2B, the fitted curve (*R*^2^ = 0.96) indicated a strong correlation between publication year and output volume. While the curve predicted 152 and 180 publications for 2023 and 2024, respectively, the actual numbers were only 108 and 93. According to Figure 2A, citation numbers increased progressively each year, attaining the highest level in 2024 (*n* = 4,482).

### 3.2 Analysis of countries and collaboration

**Figure 3:**
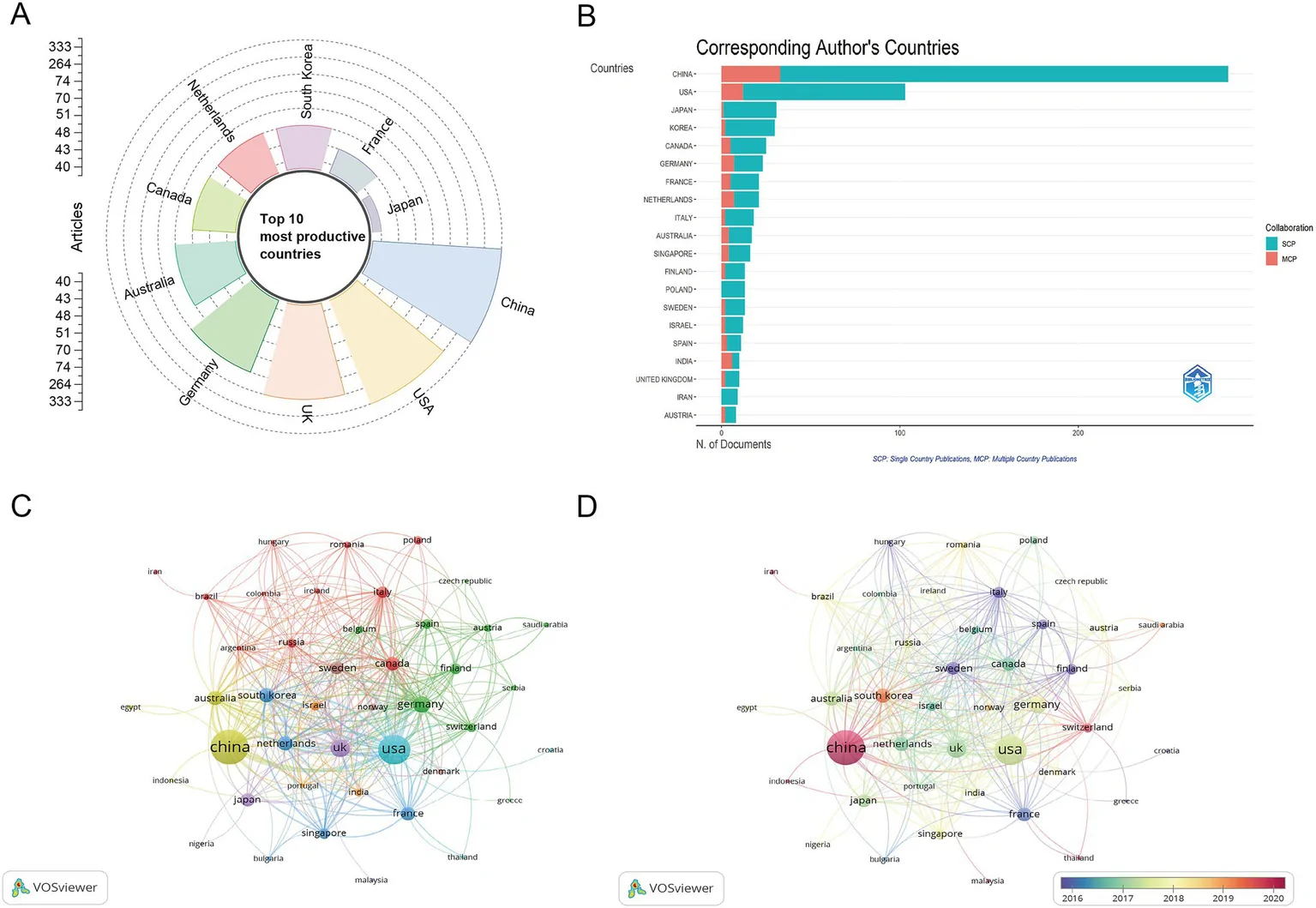
Bibliometric analysis of countries in ISCD research from 2004 to 2024. **(A)** Top 10 most productive countries. **(B)** The countries of corresponding authors. **(C)** Collaboration network of countries based on VOSviewer. Each node corresponds to a country, where larger nodes indicate greater output in this field. The connecting lines between nodes represent collaborative relationships between countries, with thicker lines denoting stronger cooperation. **(D)** Overlay visualization map of countries. Countries marked by blue and purple nodes engaged in research activities earlier upon time course before 2017, whereas countries that were highly productive in ISCD around 2018 were coded with yellow color and those became prominent around or after 2020 appeared in red.

During the analyzed timeframe, 65 countries were actively engaged in the ISCD field. To better interpret the collaborative roles of countries, we employed betweenness centrality, a metric that measures the extent to which a node serves as a bridge connecting disparate nodes within a network. A higher centrality value indicates a stronger intermediary role in linking different research groups, suggesting greater influence, while a lower value reflects a more peripheral position. As visually demonstrated in Figure 3A and Table 1, China dominated output with 333 publications (31.4% of the total), yet it exhibited limited integration within the global collaborative network (centrality = 0.04). The United States emerged as the second largest contributor (*n* = 264, 24.9% of the total), while exhibiting significantly stronger collaborative influence (centrality = 0.30). The United Kingdom ranked third with 74 papers (7.0%, 0.05). Japan occupied the tenth position, contributing 40 publications (3.8%, 0.04).

**Table 1:** Top 10 most productive countries.

Rank	Country	Articles	Proportion of the total	Centrality
1	China	333	31.4%	0.04
2	USA	264	24.9%	0.30
3	UK	74	7.0%	0.05
4	Germany	70	6.6%	0.17
5	Australia	51	4.8%	0.16
6	Canada	48	4.5%	0.08
7	Netherlands	48	4.5%	0.02
8	South Korea	48	4.5%	0.02
9	France	43	4.0%	0.04
10	Japan	40	3.8%	0.04

Figure 3B provides a detailed statistical breakdown of corresponding authors’ countries using Bibliometrix, distinguishing between collaboration types to evaluate independent research capacity versus international engagement. China commanded a substantial lead in both single country publications (SCP) and multiple country publications (MCP). However, SCP consistently outweighed MCP across all top-producing nations, suggesting stronger domestic research output relative to international collaboration.

Additionally, we employed VOSviewer software to create Figures 3C,D, showing both the network visualization and overlay mapping of global cooperation in ISCD studies. For instance, China maintained very close cooperation with the USA and Australia, with notably vibrant scientific research activities in recent years.

### 3.3 Analysis of institutions

**Figure 4:**
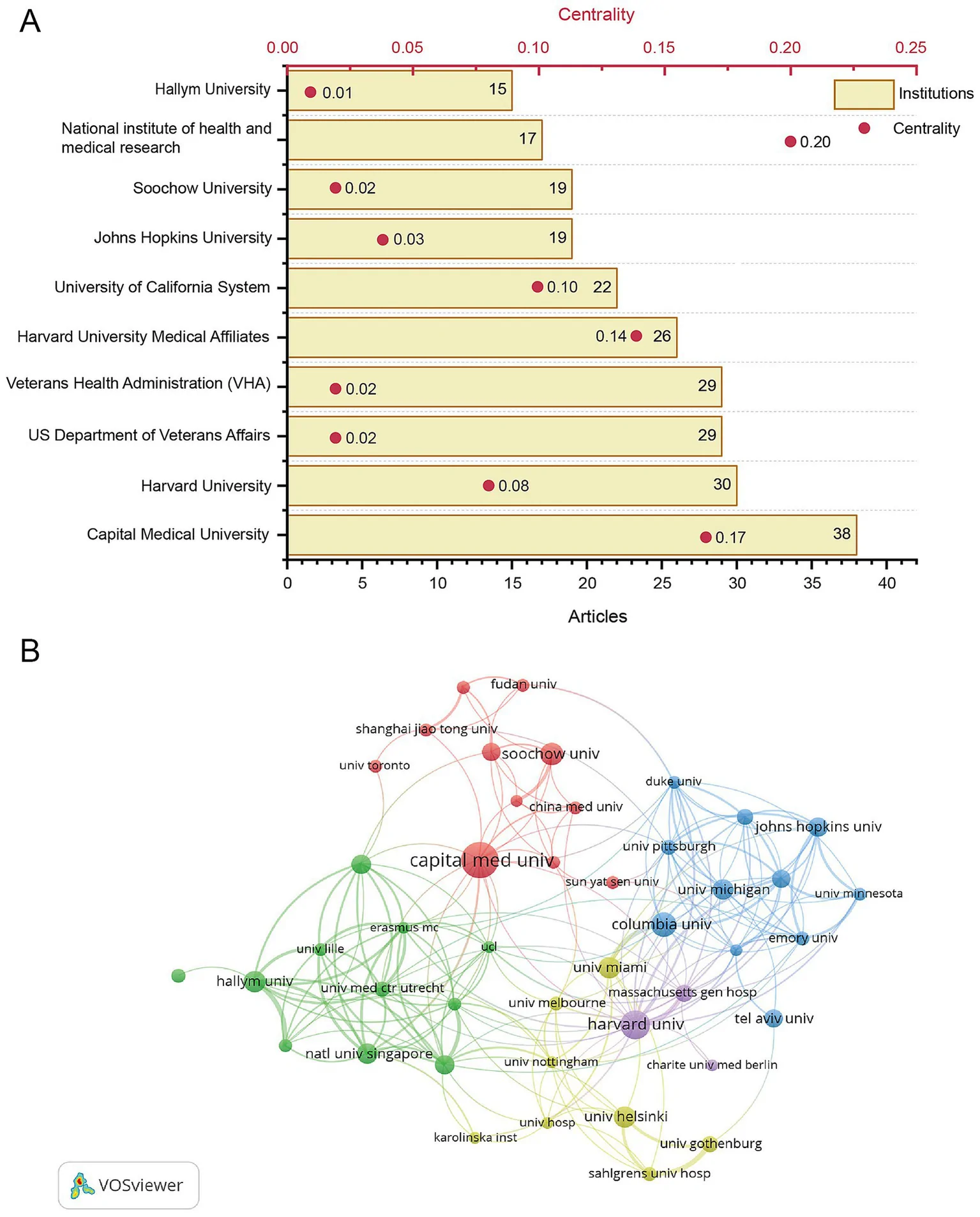
Visualization of institutions in ISCD. **(A)** The publication counts and centrality of the top 10 institutions in ISCD. **(B)** Collaboration network of institutions based on VOSviewer. Each node represents an institution. Larger nodes indicate greater output. The connecting lines between nodes represent collaborative relationships between them, with thicker lines denoting stronger cooperation.

CiteSpace-based bibliometric analysis revealed a distinct concentration of institutional productivity and centrality, as illustrated in Figure 4A. The top 10 institutions collectively contributed 22.98% of the total publications. Capital Medical University led with 38 publications (3.58%, centrality = 0.17), followed by Harvard University (*n* = 30, 2.82%, centrality = 0.08), and the US Department of Veterans Affairs (*n* = 29, 2.73%, centrality = 0.02).

To map institutional research activity and influence, we used VOSviewer to generate a co-authorship network comprising institutions with at least 10 publications. As depicted in Figure 4B, prominent institutions such as Capital Medical University, Harvard University, and Columbia University were represented by larger nodes, indicating their substantial publication output in this field. The network visualization revealed extensive inter-institutional collaboration patterns. For example, Harvard University, Columbia University, and Johns Hopkins University, as indicated by their dense interconnecting lines, formed a closely collaborative cluster.

### 3.4 Analysis of journals and co-cited journals

**Figure 5:**
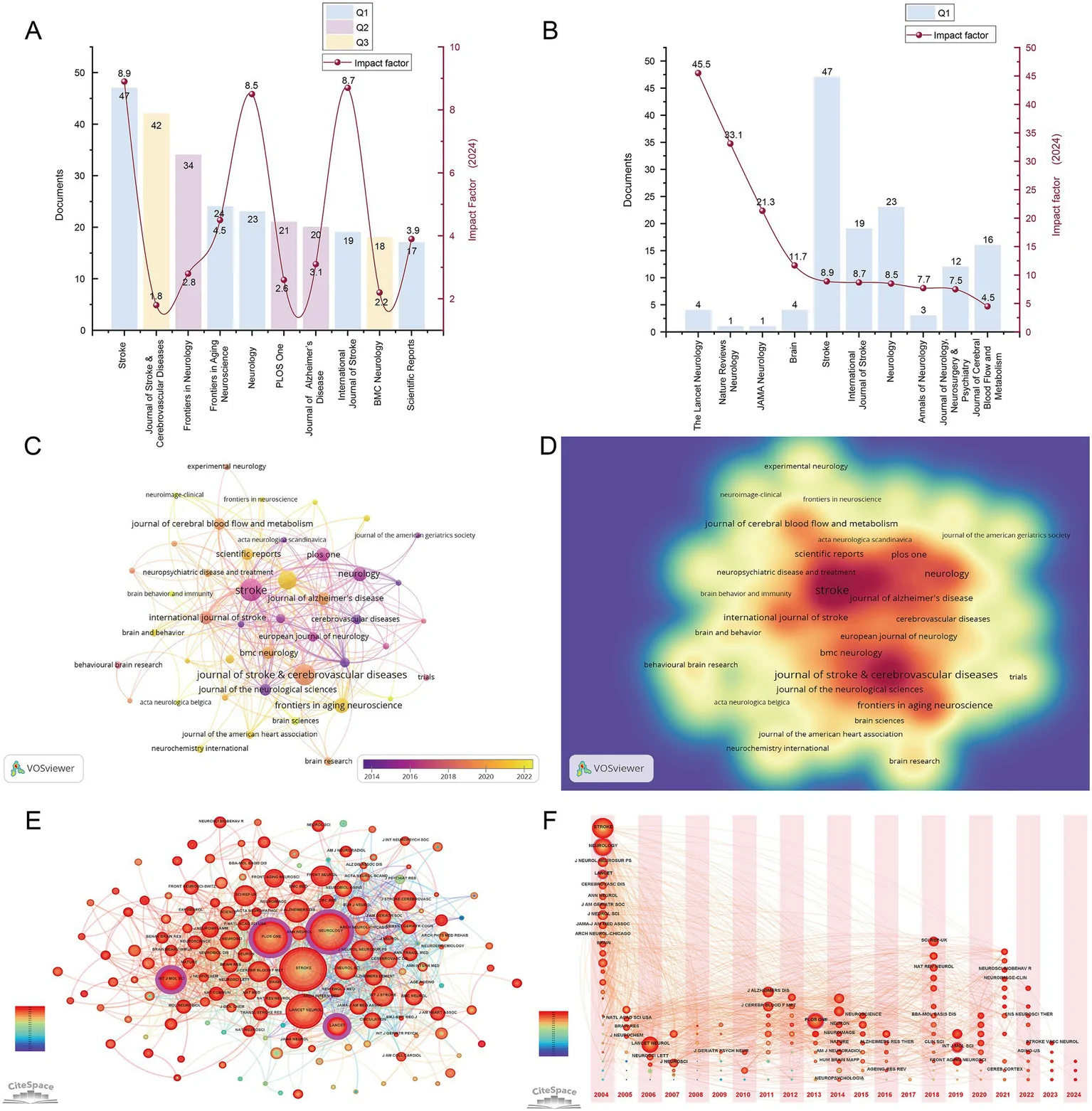
Analysis of journals and co-cited journals in ISCD. **(A)** Top 10 most productive journals and their impact factor (IF). Bar graphs with the same color represent journals with the same journal citation reports (JCR) partition. **(B)** Top 10 journals ranked by impact factor and their publications. **(C)** Overlay visualization map of journals analysis. **(D)** The density map of journals. Red areas indicate high density. The volume of ISCD studies published in journals correlating positively with both the density level and the word size. **(E)** The network of co-cited journals from CiteSpace. Where two journals are linked if they are jointly referenced by another source, forming a co-citation relationship. Journals with centrality > 0.1 will be indicated by a purple circle. **(F)** The timezone map of co-cited journals. The time zones for different journals are determined by the earliest year in which each journal was first co-cited. The color rings show how citations were distributed over time, with inner rings for early years and outer rings for recent years.

In this study, all publications came from 400 different journals. Figures 5A,B display the top 10 journals ranked by publications and impact factor (IF) in this field. *Stroke* led with 47 articles, in Journal Citation Reports (JCR) Quartile 1 (Q1), IF = 7.9, followed by *Journal of Stroke and Cerebrovascular Diseases* (*n* = 42, Q3, IF = 2), and *Frontiers in Neurology* (*n* = 34, Q2, IF = 2.7), demonstrating their high activity in ISCD research. High impact factor reflects a journal’s authoritative standing in academia (Figure 5B). *The Lancet Neurology* held the highest impact factor (Q1, 46.6), though its cumulative publication count was relatively low (*n* = 4). Similarly, *Nature Reviews Neurology* (Q1, 28.2) only published one relevant article. In contrast, *Stroke* combined high productivity (*n* = 47) with academic excellence (Q1, IF = 7.9). Overall, while both publication volume and impact factor serve as key metrics for evaluating a journal’s scholarly influence, they do not always exhibit a positive correlation.

Figures 5C,D illustrate the overlay visualization and density map of journals with at least 5 publications in ISCD. Among the top five high-output prolific journals, *Frontiers in Aging Neuroscience* was uniquely color-coded yellow, denoting both its recent publication timeframe and its ascendance as a go-to journal for cutting-edge research. *Stroke* and *Journal of Stroke & Cerebrovascular diseases* were designated as high-density areas, signifying their roles as foundational knowledge bases in this field.

We used CiteSpace to complete the visualization map of co-cited journals. As presented in Figure 5E and Table 2, *Stroke* was the most co-cited journal (co-citation = 928), followed by *Neurology* (co-citation = 692), *The Lancet Neurology* (co-citation = 528), *Journal of Neurology, Neurosurgery & Psychiatry* (co-citation = 382), and *PLOS One* (co-citation = 374). High co-citation counts highlight a journal’s foundational role in advancing the discipline. In the network of co-cited journals, *PLOS One* had the highest centrality (0.11) (nodes represented by purple circles), followed by *Neurology* (0.11), and *The Lancet* (0.11). Furthermore, there was a notable convergence between the most productive and most co-cited journals, with *Stroke*, *Neurology*, and *PLOS One* featuring in the top 10 of both categories. As shown in Figure 5F, journals are distributed chronologically (left to right) to visualize the evolution of co-citation relationships over time. The top 10 cited journals all positioned on the leftside and had wide red rings, laying the foundation for research in this field.

**Table 2:** Top 10 co-cited journals of ISCD in terms of citation count.

Rank	Co-cited journal	Citation count	Centrality	JCR	IF (2024)
1	Stroke	928	0.1	Q1	8.9
2	Neurology	692	0.11	Q1	8.5
3	The Lancet Neurology	528	0.05	Q1	45.5
4	Journal of Neurology, Neurosurgery & Psychiatry	382	0.05	Q1	7.5
5	PLOS One	374	0.13	Q2	2.6
6	The Lancet	350	0.11	Q1	88.5
7	Cerebrovascular Disease	318	0.08	Q3	1.5
8	Annals of neurology	294	0.07	Q1	7.7
9	Journal of the American Geriatrics Society	289	0.04	Q1	4.5
10	Journal of the Neurological Sciences	286	0.06	Q2	3.2

### 3.5 Analysis of authors and co-cited authors

We employed bibliometric analysis to assess researcher influence in ISCD research based on both productivity and citation metrics. Our analysis identified 6,503 contributing authors during this period. Top 10 most productive authors are listed in Table 3. Mok Vincent dominated with 16 publications, closely followed by Lim Jae Sung and Yu KH (*n* = 15). Among them, Mok Vincent had the highest centrality (0.05), while these values were all comparatively low. The h-index is a composite metric measuring both research productivity and impact. An h-index of N means that the author has N publications, each of which has received at least N citations. Among them, Sacco RL had the highest h-index (h = 134), reflecting a robust quantity-quality balance in his publication record.

**Table 3:** Top 10 productive authors referring to published articles.

Rank	Author	Articles	Centrality	Affiliation	Country	H-Index
1	Mok Vincent	16	0.05	The Chinese University of Hong Kong	China	69
2	Lim Jae Sung	15	0.01	Asan Medical Center	South Korea	28
3	Yu KH	15	0	Hallym University Sacred Heart Hospital	South Korea	41
4	Wardlaw JM	14	0.01	Ctr Univ Edinburgh	UK	126
5	Sacco RL	14	0	University of Miami	USA	134
6	Pan YS	13	0	Beijing Tiantan Hospital	China	78
7	Wang YJ	12	0	Beijing Tiantan Hospital	China	74
8	Gottesman RF	12	0	Natl Inst Neurol Disorders & Stroke Intramural Res	USA	81
9	Levine DA	11	0	University of Michigan	USA	41
10	Oh MS	11	0.02	Hallym University Sacred Heart Hospital	South Korea	34

**Figure 6:**
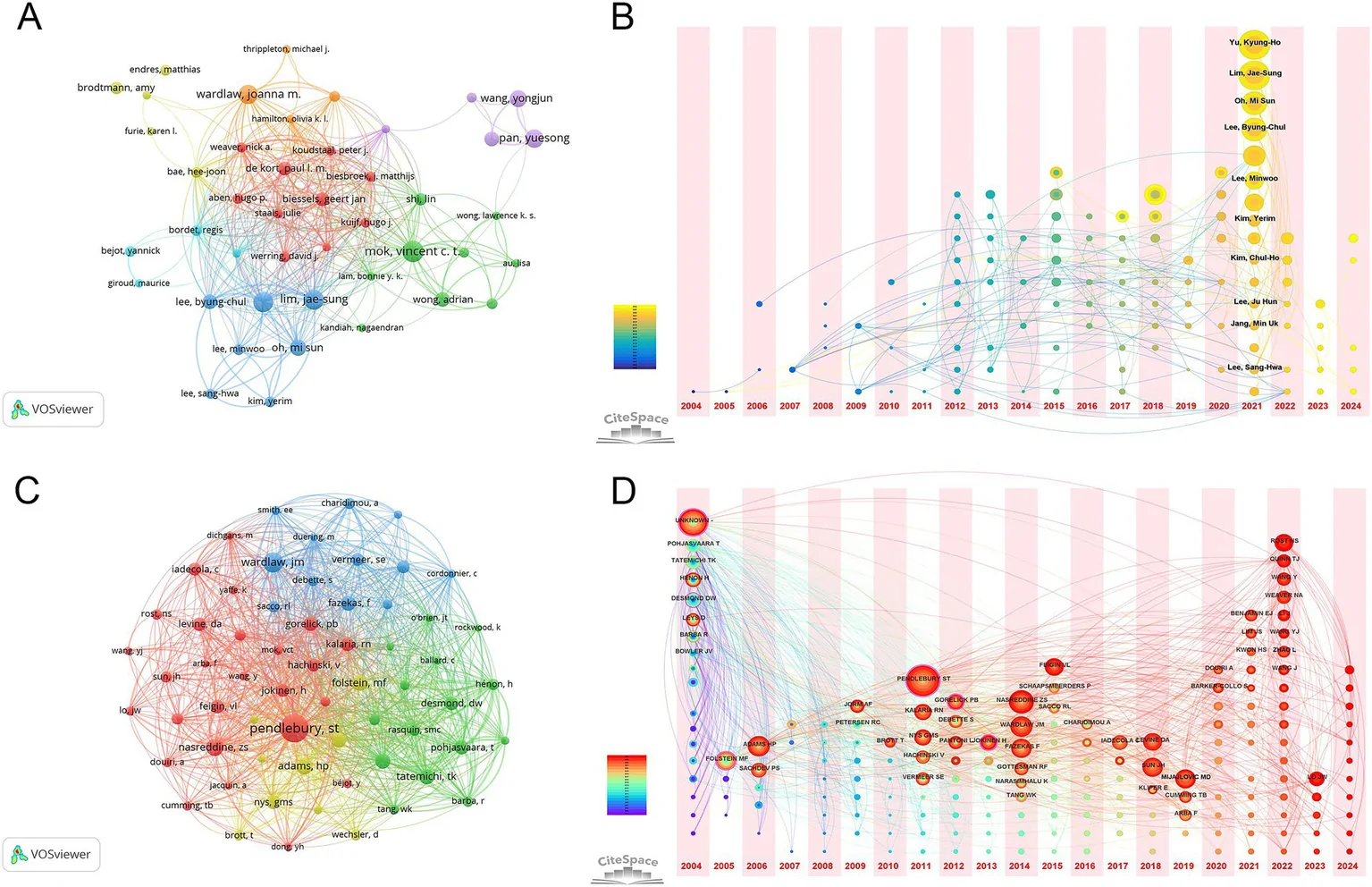
Visualization of authors and co-cited authors in ISCD. **(A)** Author’s co-authorship network (thresholds: ≥5 publications, ≥50 citations). **(B)** The time zone map of authors related to ISCD. Different authors are represented by different tree-rings, with their initial publication marking the start of rings. As time progresses, the accumulation of publications is presented by the growing size of the annual ring. There will be a connection between the two authors participating in the same article. **(C)** Co-cited network of authors. If two authors are jointly cited in the same article, a co-citation relationship between them is established. **(D)** The time zone map of co-cited authors.

Co-authorship network analysis offers a robust approach to highlight primary authors and map the density of their collaborative networks (15). We applied VOSviewer to analyze the co-authorship network (authors with at least 5 publications and 50 citations), ensuring the focus on high-impact researchers (Figure 6A). In the map, the connecting lines between nodes represent collaborative relationships among authors, with line density and thickness indicating the strength of their collaborations. The author timezone map (Figure 6B) tracks the dynamic participation of authors throughout the studied timeframe. Some productive authors such as Lim Jae Sung, Yu KH, and Oh Mi Sun began publishing articles in 2021.

It is notably shown in Figure 6C and Table 4 that Pendlebury ST (citations = 207, centrality = 0.18) had both highest citation counts and centrality, while the h-index was relatively low (h = 1). In contrast, among the top 10 co-cited authors, Wardlaw JM (citations = 94, centrality = 0.06) had the highest h-index (h = 126). We also applied CiteSpace to view the timezone map of co-cited authors. As illustrated in Figure 6D, most authors have notable visibility in recent years. Feigin VL had the widest red ring among the top 10 co-cited authors.

**Table 4:** Top 10 co-cited authors ranked by citation count.

Rank	Author	Citation count	Centrality	Affiliation	Country	H-Index
1	Pendlebury ST	207	0.18	Wolfson Ctr Prevent Stroke & Dementia	UK	1
2	Nasreddine ZS	116	0.09	MoCA Clin & Inst	Canada	27
3	Adams HP	113	0.08	The University of Iowa Healthcare	USA	29
4	Wardlaw JM	94	0.06	Ctr Univ Edinburgh	UK	126
5	Folstein MF	93	0.13	Tufts University School of Medicine	USA	69
6	Feigin VL	81	0.07	Auckland University of Technology	New Zealand	18
7	Gorelick PB	77	0.1	Northwestern University	USA	83
8	Fazekas F	74	0.05	University of Graz	Austria	104
9	Sachdev PS	73	0.08	University of New South Wales Sydney	Australia	121
10	Levine DA	65	0.05	University of Michigan	USA	41

### 3.6 Analysis of keywords

**Figure 7:**
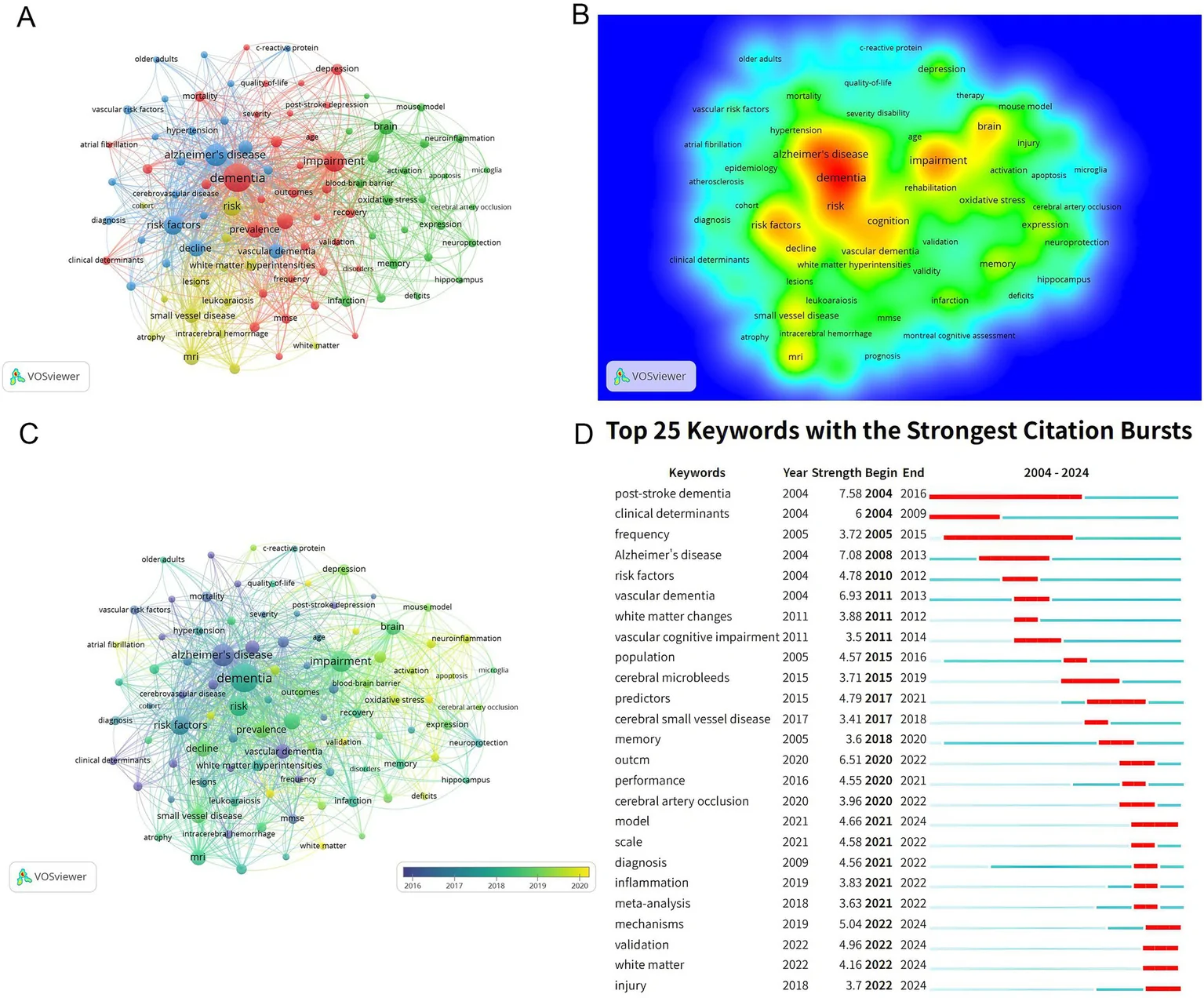
Bibliometric analysis of keywords in ISCD from 2004 to 2024. **(A)** The co-occurrence network of keywords (≥15 occurrences). The 125 keywords were divided into four distinct clusters, each represented by a unique color (red, green, yellow, and blue). The node size indicates the occurrence frequency. Lines between nodes express association between them. **(B)** The density map of keywords. Warm colors refer to areas with highly concentrated research, while cool colors indicate relatively understudied areas. **(C)** The overlay visualization map of keywords. The nodes coded with purple and blue color represented the keywords that appeared relatively earlier upon time course before or around 2017, whereas keywords that appeared around 2019 were coded with green color and those frequently used around or after 2020 appeared in yellow. **(D)** Keywords with the strongest citation bursts in ISCD (sorted by the beginning year of burst). The red bands stand for burst duration periods, while light blue denotes time spans before keyword emergence and dark blue represents appearance periods of keywords.

Our study analyzed a total of 4,228 keywords, with 125 meeting the frequency threshold (at least 15 occurrences) for visualization in VOSviewer to ensure analytical relevance. As shown in Figure 7A and Supplementary Table 3, keywords including “dementia” (218 occurrences, 0.17), “Alzheimer’s disease” (177 occurrences, 0.22), “impairment” (150 occurrences, 0.10), “risk” (138 occurrences, 0.07), and “risk factors” (127 occurrences, 0.09) appeared as significantly larger nodes with warm color in the density map (Figure 7B), indicating their status as critically important core keywords that fundamentally define the research themes.

We used both VOSviewer and CiteSpace to analyze the emergence and burst of keywords, as illustrated in Figures 7C,D. In bibliometric analysis, a “citation burst” refers to a sharp, statistically significant surge in the occurrence frequency of a keyword or reference over a defined period, indicating a sudden rise in scholarly interest. We applied this metric to identify emerging research hotspots, track thematic shifts over time, and gain deeper insights into the evolving dynamics of ISCD. For each keyword, CiteSpace calculates a burst strength value that quantifies the relative increase in frequency during the burst period against the baseline level. We identified the top 25 keywords with the strongest citation bursts, represented chronologically with blue time-interval lines and red burst-intensity bars. Post-stroke dementia demonstrated the highest burst strength (7.58), followed by Alzheimer’s disease (7.08), and vascular dementia (6.93). Based on Figure 7D, the temporal distribution of keyword bursts indicates that, in the early phase, studies primarily focused on “post-stroke dementia,” “Alzheimer’s disease,” investigating the association between ischemic stroke-specific cognitive dysfunction and classical dementia diseases. With the emergence of keywords bursts in 2011, such as “vascular dementia,” “vascular cognitive impairment,” and “white matter changes,” research focus shifted toward cerebrovascular pathologies and factors related to brain structure. In recent years, keywords such as “model,” “scale,” and “validation” have frequently appeared. Additionally, the bursts of “mechanisms” and “inflammation” indicate that inflammatory mechanism has become a research hotspot.

### 3.7 Analysis of references

To identify references with the most extensive influence in ISCD, we utilized Bibliometrix to extract the top 10 most globally cited documents. As listed in Supplementary Table 4, the study published in *The Lancet Neurology*, written by Pendlebury ST et al. (16), led with 1,233 citation counts (Q1, IF = 45.5), systematically elucidating the epidemiology, incidence, and key risk factors associated with both pre-stroke and post-stroke dementia. Other highly cited studies included Vernooij MW et al. (17) (citation counts = 636), Psaltopoulou T et al. (18) (citation counts = 572), and Newman CB et al. (19) (citation counts = 479).

**Figure 8:**
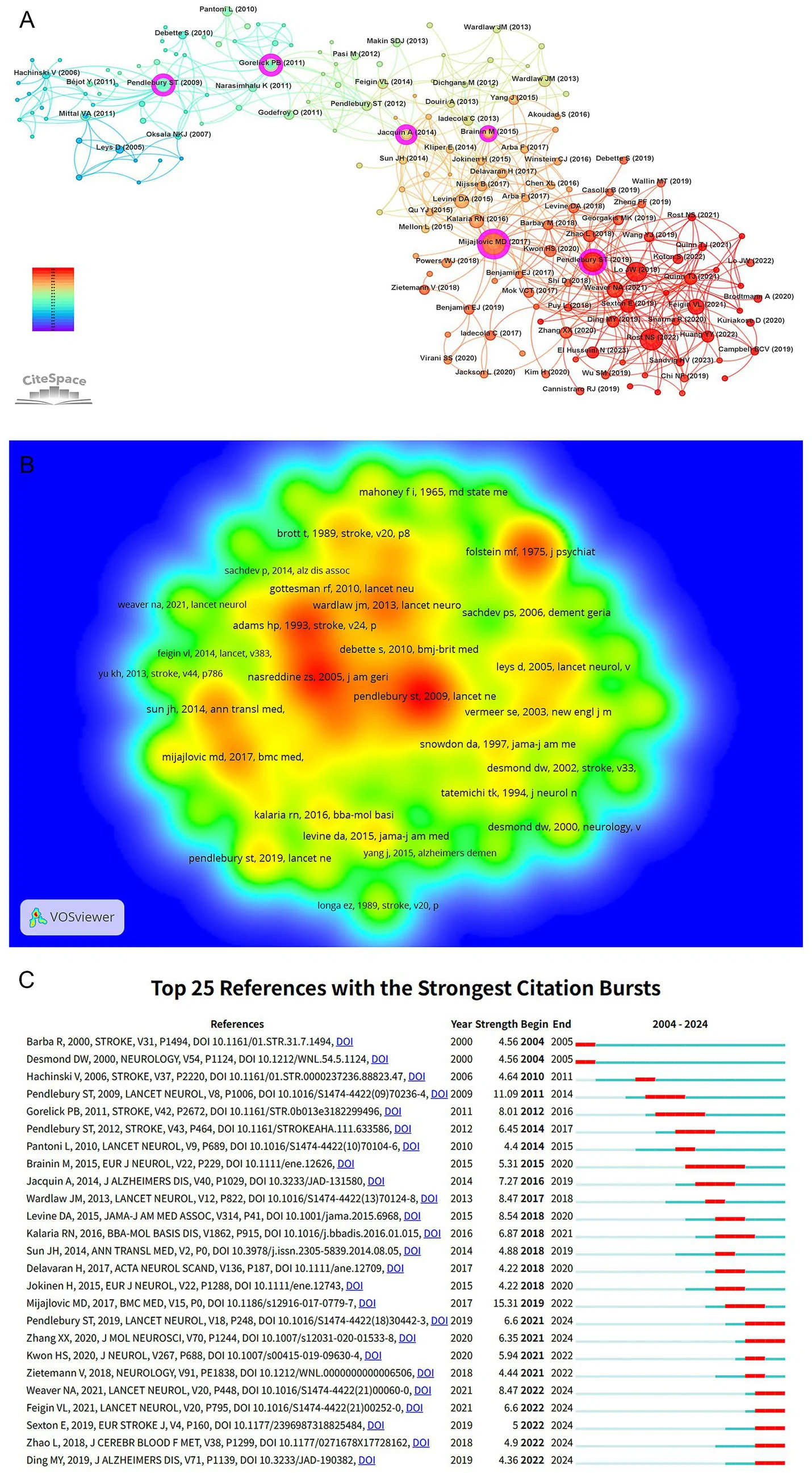
Visualization analysis of co-cited references. **(A)** Co-citation network of references from 2004. **(B)** The density map of co-cited references. **(C)** Top 25 references with the strongest citation burst (sorted by the beginning year of burst).

To further reveal the intellectual base structure underpinning this research domain and the evolutionary dynamics of research themes, we applied CiteSpace to conduct co-citation analysis of references. A co-citation relationship is established if two references are cited together in a third article. A high co-citation count indicates that the reference’s content is regarded as foundational knowledge in the field. A high centrality signifies that the reference serves as the nucleus of attention, spawning derivative publications. Among the top 10 frequently co-cited references (Supplementary Table 5), four were published in *The Lancet Neurology*, and two of these were authored by Pendlebury ST. As shown in Figure 8A, the study by Mijajlović MD et al. (20) and Pendlebury ST et al. (21) appeared as most visually prominent large nodes in the map with purple circles, indicating that they attracted significant scholarly attention. Figure 8B also demonstrates that the studies by Pendlebury ST et al. (16) and Nasreddine ZS et al. (22) are widely recognized consensual papers in this field.

We used CiteSpace to identify the top 25 references with the strongest citation bursts (Figure 8C). It is noteworthy that the study by Mijajlović et al. (20) exhibited the strongest citation burst strength (15.31), elucidating the definitions, epidemiological profile, assessment tools, and diagnostic challenges of post-stroke cognitive impairment (PSCI) and post-stroke dementia (PSD), and providing essential foundations for emerging research in this field. Moreover, recent research including those by Zhao et al. (23), Zietemann et al. (24), Ding et al. (25), and Weaver et al. (26) has pivoted toward utilizing different assessment and predictive tools and developing relevant models, enabling deeper insights into ISCD.

### 3.8 Analysis of inflammation related researches

**Figure 9:**
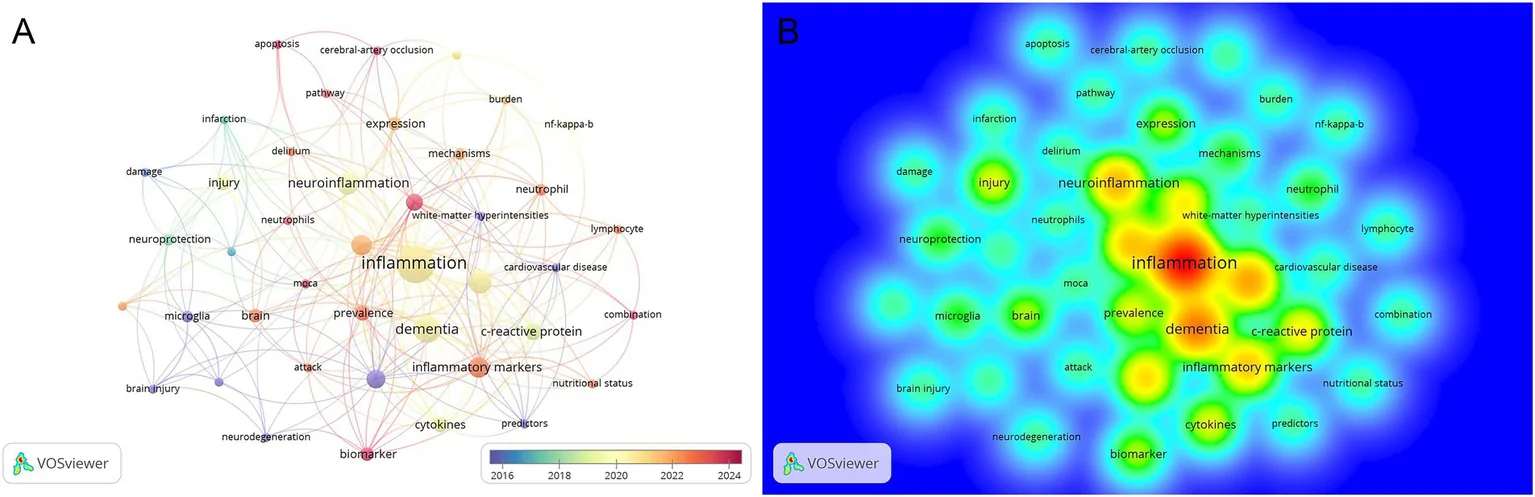
Visualization analysis of keywords in inflammation research related to ISCD. **(A)** The overlay visualization map of keywords. **(B)** The density visualization map of keywords.

In this study, we noticed that the keywords “inflammation” and “mechanisms” began to emerge and burst in 2019. The inflammatory response following ischemic stroke leads to blood–brain barrier disruption and neuronal damage, consequently impairing cognitive function (5). We retrieved 42 inflammation-related articles from the 1,062 original studies and separately imported them into VOSviewer for keyword co-occurrence analysis (Figure 9). The top 10 most frequent keywords are listed in Table 5. “dementia” (11, 0.41) emerged as the highest-frequency keyword, followed by “inflammatory markers” (8, 0.10). Notably, inflammatory markers, particularly biomarkers such as “C-reactive protein” (4, 0.09), have garnered particular research attention.

**Table 5:** Top 10 most frequent keywords in inflammation related to ISCD.

Rank	Words	Occurrences	Centrality
1	dementia	11	0.41
2	inflammatory markers	8	0.10
3	decline	8	0.24
4	inflammation	7	0.24
5	risk factors	7	0.05
6	Alzheimer’s disease	5	0.23
7	brain	4	0.07
8	expression	4	0.16
9	injury	4	0.17
10	C-reactive protein	4	0.09

## 4 Discussion

### 4.1 General information and evolving trends in ISCD research

Our bibliometric analysis delineates the scientific landscape of ISCD research from 2004 to 2024. The field has demonstrated substantial growth. Annual publications exceeded 100 in 2021 and reached 133 by 2022 (Figure 2A). This steady upward trajectory may be partly attributable to the escalating recognition of the clinical burden of ISCD and the maturation of dedicated research initiatives (27). In parallel, citation counts have increased progressively, reaching their highest level in 2024 (Figure 2A), indicating sustained scientific impact despite fluctuations in publication volume. However, the actual publications in 2023 and 2024 (108 and 93) were markedly lower than growth curve predictions of 152 and 180 (Figure 2B). This deviation may stem from the staged development cycles that research fields commonly undergo (28). Additionally, because the data were collected on February 7, 2025, publications from recent years may be subject to incomplete database coverage, a well-documented phenomenon in bibliometric studies (29).

Beyond quantitative growth, the thematic focus of ISCD research has also undergone a notable evolution. The keyword burst timeline analysis (Figure 7D) shows that, compared with earlier studies that often addressed ISCD alongside other conditions such as Alzheimer’s disease (AD), vascular dementia (VaD), and vascular cognitive impairment (VCI) in the past, research since 2020 has shifted the focus to ISCD as the primary subject, investigating its clinical performance, outcome, and mechanisms.

China and the USA lead in research productivity but exhibit distinct collaborative strategies. China’s high output yet low centrality indicates a focus on domestic research or limited networks, while the USA’s high centrality positions it as a global hub. Strengthening international partnerships could boost China’s research impact. Among numerous institutions, Capital Medical University emerged as the most significant contributor. *Stroke* is the journal with the largest number of publications and co-citations, demonstrating its high academic level and influence.

### 4.2 Key themes and research hotspots analysis

#### 4.2.1 The emerging frontier of inflammatory markers of ISCD

“Risk factors” has consistently appeared as a keyword from 2004 to 2024 (Figure 7D), reflecting its enduring status as a research priority in this field, as evidenced by previous bibliometric analyses of PSCI (30, 31). The pathophysiology of ischemic stroke is a complex, inflammation-involved process that not only causes vascular damage but also harms brain tissue, thereby increasing the risk of cognitive impairment (32). Notably, subgroup analyses in our study on inflammation confirmed that inflammatory markers are key risk factors for ISCD (Figure 9).

##### 4.2.1.1 Pro-inflammatory cytokines

The tight coupling between “cytokines” and the core node “inflammation” in our co-occurrence network (Figure 9A) highlights cytokines-related research as a major subtopic in ISCD research. It is well-established that pro-inflammatory cytokines serve as key mediators of neuroinflammation (33). Among the diverse cytokine families, interleukins, particularly IL-6, IL-8, and IL-12, are involved in signal transduction, immunomodulation, and inflammatory responses, with their levels elevated during central nervous system injury or infection (34). A prospective cohort study of 223 IS patients confirmed that elevated serum IL-6 levels in the acute phase were significantly correlated with MoCA scores and independently associated with cognitive impairment (35). Narasimhalu et al. demonstrated temporal specificity of interleukin effects: higher IL-8 is independently associated with baseline cognitive impairment, whereas higher serum IL-12 is associated with subsequent cognitive decline after IS (36). Beyond the interleukin family, Tumor Necrosis Factor-α (TNF-α) is likewise a classical pro-inflammatory cytokine. Loga-Andrijic et al. found that elevated levels of IL-6 and TNF-α are significantly associated with cognitive impairment in patients with acute ischemic stroke (AIS), with notable sex-specific differences (37). Specifically, cognitive impairment in female patients is more strongly linked to TNF-α-driven inflammation, which exhibits sustained effects, whereas cognitive decline in male patients may depend primarily on the acute-phase role of IL-6.

##### 4.2.1.2 C-reactive protein

According to Table 5, the high frequency of the keyword “C-reactive protein” underscores its pivotal role in inflammation-associated studies in ISCD. C-reactive protein (CRP) is a liver-derived plasma protein that serves as a common but nonspecific inflammatory marker, with higher concentrations correlating with increased risks of cerebrovascular disorders and cognitive decline. Irimie et al. found that plasma CRP levels (*β* = 0.457, *p* < 0.001) significantly predicted poor functional outcomes in AIS patients (38). Cai et al. reported that elevated plasma high-sensitivity C-reactive protein (hs-CRP) levels were associated with increased risk of neurological deterioration and poor prognosis (*p* < 0.05) (39).

##### 4.2.1.3 Composite inflammatory indices

The prominence of “neutrophil” and “lymphocyte” as distinct nodes in the keyword co-occurrence map (Figures 9A,B) points to the widespread interest in composite inflammatory indices integrating these two cell types. Neutrophil-to-lymphocyte ratio (NLR) is a well-validated composite index that quantifies systemic inflammatory burden. In a prospective cohort study of 224 patients, Zhao et al. found that elevated NLR was significantly associated with an increased risk of PSCI at 6–12 months of follow-up. Notably, incorporating NLR into predictive models that already included demographic, clinical, and imaging information significantly improved the area under the curve (AUC) from 0.765 to 0.803 (*p* = 0.049), underscoring its incremental prognostic value beyond conventional risk factors (40).

##### 4.2.1.4 Novel composite biomarkers

While conventional inflammatory markers dominate the core keyword clusters (Figures 9A,B), co-occurrence of “biomarker,” “nutritional status” and “predictors” indicates a growing trend toward incorporating inflammatory and nutritional variables for ISCD risk stratification. The lactate dehydrogenase to albumin ratio (LAR) is a novel composite biomarker that integrates markers of cellular injury (lactate dehydrogenase, LDH) and nutritional status (albumin), thereby reflecting both systemic inflammation and protein nutrition. Xu et al. measured LDH and albumin levels in AIS patients within 24 h after hospitalization (41). They found that LAR levels were significantly higher in AIS patients with PSCI than in those without PSCI (*p* < 0.001), demonstrating that LAR is independently associated with acute-phase cognitive decline. Similarly, the HALP score, which integrates hemoglobin, albumin, lymphocyte, and platelet counts, has been found to correlate with ISCD. Lower HALP scores in AIS patients were significantly associated with the presence of PSCI (42, 43).

##### 4.2.1.5 Assessment tools and prediction models of ISCD

The emergence and prominence of keywords such as “model,” “validation,” and “scale” in recent years (Figure 7D) reflect growing interest in multidisciplinary approaches to ISCD research. Several factors may explain this trend: (1) the growing availability of large-scale clinical datasets facilitates data-driven modeling (44); (2) advances in artificial intelligence, including machine learning and deep learning, enable integration of multidimensional data beyond traditional regression approaches (45); (3) the multifactorial nature of ISCD demands multidisciplinary collaboration (46); and (4) the shift toward precision medicine drives demand for individualized risk prediction tools to guide personalized care (47).

**Figure 10:**
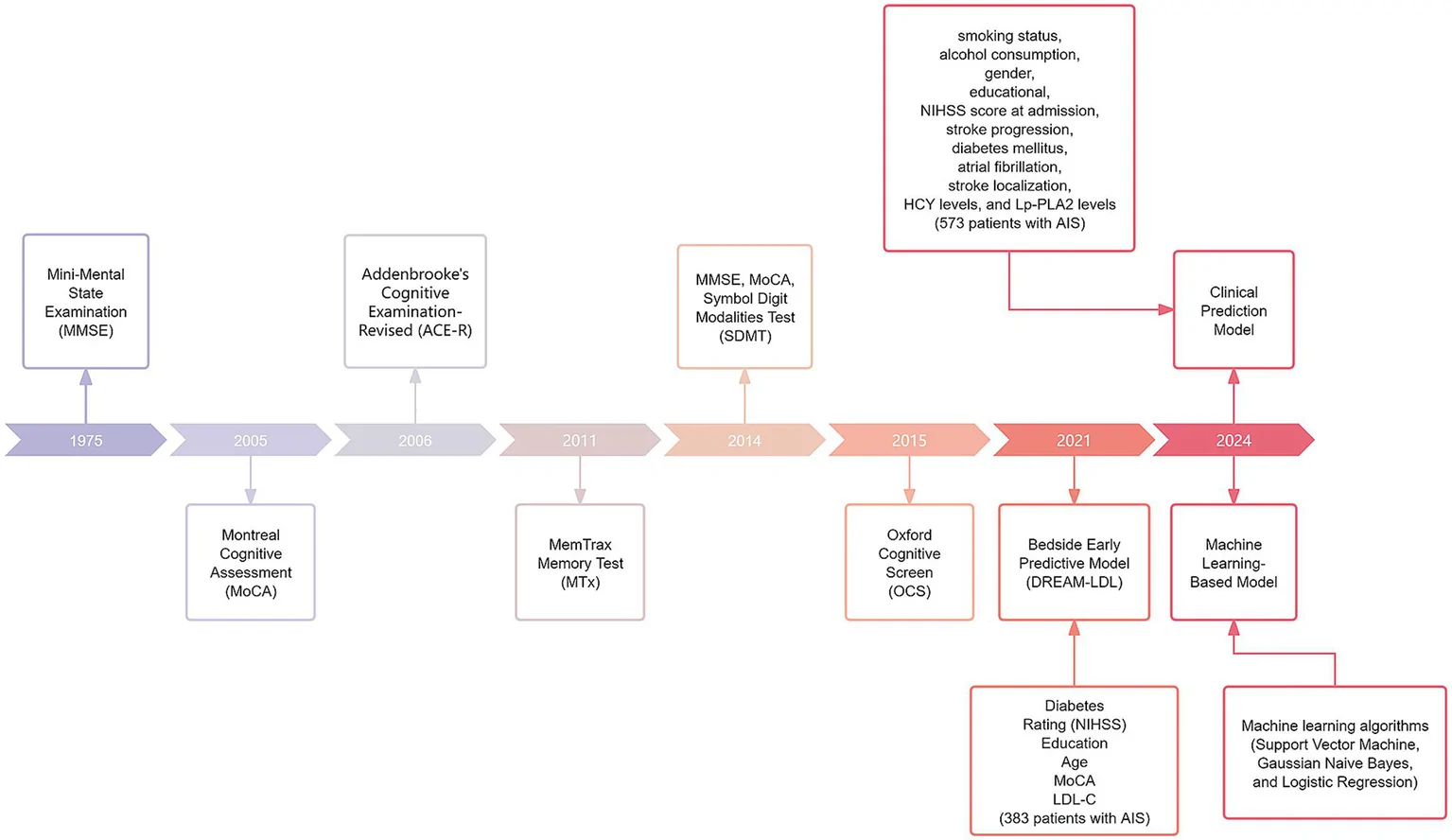
Evolution of cognitive assessment tools and prediction models for ISCD.

The application of appropriate cognitive assessment instruments is fundamental for both outcome definition in model development and screening in clinical practice. Several widely used cognitive screening tools are based on behavioral performance. As illustrated in Figure 10, the Mini-Mental State Examination (MMSE, 1975) and the Montreal Cognitive Assessment (MoCA, 2005) remain common in clinical practice (22, 48). A recent meta-analysis found no significant difference between MoCA and MMSE in detecting cognitive impairment after IS (pooled sensitivity 0.80 vs. 0.76, specificity 0.79 vs. 0.78), recommending both be considered based on clinical purpose and feasibility (49). Furthermore, adding the Symbol Digit Modalities Test (SDMT) to either MoCA or MMSE improves screening accuracy for VCI in IS patients (50). Other instruments, including the Addenbrooke’s Cognitive Examination-Revised (ACE-R) and the MemTrax Memory Test (MTx), also serve as screening tools (51). Compared with traditional tools, MTx has been demonstrated as a rapid (2-min) and effective (AUC = 0.92) screening tool in a validation study of 104 patients assessed 90 days after AIS (52). Notably, the stroke-specific Oxford Cognitive Screen (OCS), developed in 2015, has shown greater promise (53). A prospective cohort study demonstrated that the OCS administered acutely predicts long-term functional outcomes at 6 and 12 months, accounting for 61–79% of variance across multiple SIS 3.0 domains—substantially outperforming demographics and the NIHSS (54). Collectively, these refinements of assessment instruments have enhanced screening sensitivity and feasibility, strengthening the foundation for predictive modeling.

Based on advances in cognitive assessment, several studies have integrated clinical variables to predict cognitive impairment after AIS. Compared with traditional observational studies that primarily identify associations between individual risk factors and cognitive outcomes, prediction models offer a more integrated and clinically actionable framework by quantifying the combined predictive value of multiple variables at the individual patient level. For example, Dong et al. developed a clinical model (DREAM-LDL) using multivariate regression incorporating diabetes (fasting blood glucose level), NIHSS score, education level, age, baseline MoCA, and low-density lipoprotein cholesterol (LDL-C) level to predict the occurrence of cognitive impairment at 6 months after AIS (55). However, its retrospective design and lack of external validation limit its generalizability. Wei et al. further constructed a comprehensive logistic regression model in 573 AIS patients across development, internal validation, and external validation cohorts, demonstrating robust predictive performance (AUC = 0.847–0.898). Notably, the inclusion of an external validation cohort enhanced the model’s generalizability across different populations (56). More recently, Zhang et al. applied three common machine learning algorithms to build clinical prediction models of cognitive impairment in AIS, including support vector machine, Gaussian Naive Bayes, and logistic regression (57). The logistic regression model demonstrated high performance (sensitivity = 0.82, specificity = 0.83, accuracy = 0.83). This study explored the feasibility of machine learning approaches for ISCD prediction, though its cross-sectional design and lack of external validation limit broader applicability.

These examples illustrate how prediction models have begun to improve risk stratification by enabling clinicians to identify high-risk patients who may benefit from more intensive follow-up or early intervention. However, the current evidence base has notable gaps: most models remain at the development or internal validation stage, and few have undergone external validation in independent cohorts or prospective implementation studies. Future efforts should prioritize multicenter external validation and prospective implementation studies to bridge this gap and translate model performance into tangible clinical benefit.

## 5 Limitation

In our study, several limitations should be acknowledged. Although we performed a cross-validation analysis by comparing WoSCC with PubMed and confirmed complete literature concordance for our search terms and time frame, we cannot rule out the possibility that relevant publications indexed exclusively in other databases (e.g., Scopus, Embase, or regional databases) might have been missed. Moreover, because the database is continuously updated, the retrieved results reflect the snapshot at the time of our search (conducted on February 7, 2025), and minor discrepancies may occur if the same search were repeated at a later date.

We restricted inclusion to English-language publications. While it is a common practice in bibliometric studies to ensure consistency in indexing and citation data, this criterion may have introduced geographic and linguistic biases, potentially overlooking research from non-English regions and artificially elevating the visibility of English-speaking countries, institutions, and authors.

Furthermore, bibliometric analysis is inherently descriptive rather than evaluative. It can map publication trends, citation patterns, and collaborative relationships, but it does not substitute for critical appraisal of study methodology, validity of conclusions, or clinical relevance.

## 6 Conclusion

Our study conducted a bibliometric analysis of publications in the field of ischemic stroke-specific cognitive dysfunction (ISCD) from 2004 to 2024. The results demonstrated a significant upward trend in annual publication output. China and the United States emerged as the leading contributors to research productivity. Single-country publications predominated over internationally collaborative works, implying that Strengthened international cooperation is urgently needed to advance the field.

The identified keyword clusters and hot topics further characterize the current research priorities of ISCD, which mainly focus on inflammatory biomarkers, cognitive assessment tools, and predictive modeling. This thematic shift demonstrates that the field is gradually moving from traditional clinical observation toward precise risk stratification and translational biomarker research.

Therefore, future research should place greater emphasis on multicenter prospective validation of existing prediction models, integration of multi-omics and biomarkers for enhanced mechanistic interpretability, with the goal of ultimately advancing clinical translation.

## Data Availability

The datasets presented in this study can be found in online repositories. The names of the repository/repositories and accession number(s) can be found in the article/Supplementary material.

## References

[1] Hilkens NA Casolla B Leung TW de Leeuw F-E. Stroke. Lancet. (2024) 403:2820–36. doi: 10.1016/S0140-6736(24)00642-1, 38759664

[2] Furie KL Kelly PJ. Secondary prevention after ischemic stroke. N Engl J Med. (2026) 394:784–92. doi: 10.1056/NEJMcp2415601, 41707139

[3] Filler J Georgakis MK Dichgans M. Risk factors for cognitive impairment and dementia after stroke: a systematic review and meta-analysis. Lancet Healthy Longev. (2024) 5:e31–44. doi: 10.1016/S2666-7568(23)00217-9, 38101426

[4] Eltzschig HK Eckle T. Ischemia and reperfusion--from mechanism to translation. Nat Med. (2011) 17:1391–401. doi: 10.1038/nm.2507, 22064429 PMC3886192

[5] DeLong JH Ohashi SN O’Connor KC Sansing LH. Inflammatory responses after ischemic stroke. Semin Immunopathol. (2022) 44:625–48. doi: 10.1007/s00281-022-00943-7, 35767089

[6] Zhang M Liu Q Meng H Duan H Liu X Wu J . Ischemia-reperfusion injury: molecular mechanisms and therapeutic targets. Signal Transduct Target Ther. (2024) 9:12. doi: 10.1038/s41392-023-01688-x, 38185705 PMC10772178

[7] Zhang L Meng F Ge R Tian X Sun P Wang Z . The bibliometric and visualized analysis on cancer stem cells in the early 21st century. Int J Surg. (2026) 112:1465–78. doi: 10.1097/JS9.0000000000003368, 40968763 PMC12825834

[8] Abumalloh RA Nilashi M Samad S Ahmadi H Alghamdi A Alrizq M . Parkinson’s disease diagnosis using deep learning: a bibliometric analysis and literature review. Ageing Res Rev. (2024) 96:102285. doi: 10.1016/j.arr.2024.102285, 38554785

[9] Yang Z Hotterbeex P Marent P-J Cerin E Thomis M van Uffelen J. Physical activity, sedentary behaviour, and cognitive function among older adults: a bibliometric analysis from 2004 to 2024. Ageing Res Rev. (2024) 97:102283. doi: 10.1016/j.arr.2024.102283, 38552882

[10] Zhang Q Zeng Y Zheng S Chen L Liu H Chen H . Research hotspots and frontiers of stem cells in stroke: a bibliometric analysis from 2004 to 2022. Front Pharmacol. (2023) 14:1111815. doi: 10.3389/fphar.2023.1111815 PMC10020355 36937837

[11] Wang C Gu L Zhang Y Gao Y Jian Z Xiong X. Bibliometric insights into the inflammation and mitochondrial stress in ischemic stroke. Exp Neurol. (2024) 378:114845. doi: 10.1016/j.expneurol.2024.114845, 38838802

[12] Wei N Xu Y Li Y Shi J Zhang X You Y . A bibliometric analysis of T cell and atherosclerosis. Front Immunol. (2022) 13:948314. doi: 10.3389/fimmu.2022.948314, 36311729 PMC9606647

[13] Chen C Hu Z Liu S Tseng H. Emerging trends in regenerative medicine: a scientometric analysis in CiteSpace. Expert Opin Biol Ther. (2012) 12:593–608. doi: 10.1517/14712598.2012.674507, 22443895

[14] Arruda H Silva ER Lessa M Proença DJ Bartholo R. VOSviewer and Bibliometrix. J Med Libr Assoc. (2022) 110:392–5. doi: 10.5195/jmla.2022.1434, 36589296 PMC9782747

[15] Wang R Zhu Y Qin L-F Xu Z-G Gao X-R Liu C-B . Comprehensive bibliometric analysis of stem cell research in Alzheimer’s disease from 2004 to 2022. Dement Geriatr Cogn Disord. (2023) 52:47–73. doi: 10.1159/000528886, 37068473

[16] Pendlebury ST Rothwell PM. Prevalence, incidence, and factors associated with pre-stroke and post-stroke dementia: a systematic review and meta-analysis. Lancet Neurol. (2009) 8:1006–18. doi: 10.1016/S1474-4422(09)70236-4, 19782001

[17] Vernooij MW van der Lugt A Ikram MA Wielopolski PA Niessen WJ Hofman A . Prevalence and risk factors of cerebral microbleeds - the Rotterdam scan Study. Neurology. (2008) 70:1208–14. doi: 10.1212/01.wnl.0000307750.41970.d9, 18378884

[18] Psaltopoulou T Sergentanis TN Panagiotakos DB Sergentanis IN Kosti R Scarmeas N. Mediterranean diet, stroke, cognitive impairment, and depression: a meta-analysis. Ann Neurol. (2013) 74:580–91. doi: 10.1002/ana.23944, 23720230

[19] Newman CB Preiss D Tobert JA Jacobson TA Page RL II Goldstein LB . Statin safety and associated adverse events: a scientific statement from the American Heart Association. Arterioscler Thromb Vasc Biol. (2019) 39:E38–81. doi: 10.1161/ATV.0000000000000073 30580575

[20] Mijajlovic MD Pavlovic A Brainin M Heiss W-D Quinn TJ Ihle-Hansen HB . Post-stroke dementia - a comprehensive review. BMC Med. (2017) 15:11. doi: 10.1186/s12916-017-0779-7 PMC5241961 28095900

[21] Pendlebury ST Rothwell PM Study OV. Incidence and prevalence of dementia associated with transient ischaemic attack and stroke: analysis of the population-based Oxford vascular Study. Lancet Neurol. (2019) 18:248–58. doi: 10.1016/S1474-4422(18)30442-3 PMC6390174 30784556

[22] Nasreddine Z Phillips N Bédirian V Charbonneau S Whitehead V Collin I . The Montreal cognitive assessment, MoCA: a brief screening tool for mild cognitive impairment. J Am Geriatr Soc. (2005) 53:695–9. doi: 10.1111/j.1532-5415.2005.53221.x, 15817019

[23] Zhao L Biesbroek JM Shi L Liu W Kuijf HJ Chu WWC . Strategic infarct location for post-stroke cognitive impairment: a multivariate lesion-symptom mapping study. J Cereb Blood Flow Metab. (2018) 38:1299–311. doi: 10.1177/0271678X17728162, 28895445 PMC6092771

[24] Zietemann V Georgakis MK Dondaine T Mueller C Mendyk A-M Kopczak A . Early MoCA predicts long-term cognitive and functional outcome and mortality after stroke. Neurology. (2018) 91:E1838–50. doi: 10.1212/WNL.0000000000006506 30333158

[25] Ding M-Y Xu Y Wang Y-Z Li P-X Mao Y-T Yu J-T . Predictors of cognitive impairment after stroke: a prospective stroke cohort study. J Alzheimer's Dis. (2019) 71:1139–51. doi: 10.3233/JAD-190382 31524163

[26] Weaver NA Kuijf HJ Aben HP Abrigo J Bae H-J Barbay M . Strategic infarct locations for post-stroke cognitive impairment: a pooled analysis of individual patient data from 12 acute ischaemic stroke cohorts. Lancet Neurol. (2021) 20:448–59. doi: 10.1016/S1474-4422(21)00060-0, 33901427

[27] Huang Y-Y Chen S-D Leng X-Y Kuo K Wang Z-T Cui M . Post-stroke cognitive impairment: epidemiology, risk factors, and management. J Alzheimer's Dis. (2022) 86:983–99. doi: 10.3233/JAD-215644, 35147548

[28] Singh CK Barme E Ward R Tupikina L Santolini M. Quantifying the rise and fall of scientific fields. PLoS One. (2022) 17:e0270131. doi: 10.1371/journal.pone.0270131, 35737658 PMC9223313

[29] Zhu Y. Evaluation methods and empirical research on comprehensiveness, accuracy and timeliness of early access indexing in the web of science. J Inf Sci. (2025). doi: 10.1177/01655515251379429

[30] Chi X Fan X Fu G Liu Y Zhang Y Shen W. Research trends and hotspots of post-stroke cognitive impairment: a bibliometric analysis. Front Pharmacol. (2023) 14:1184830. doi: 10.3389/fphar.2023.1184830, 37324494 PMC10267734

[31] Zhang H Li Y Zhan L Long J Shen J Chen J . Knowledge domain and emerging trends in post-stroke cognitive impairment: a bibliometric analysis. Front Aging Neurosci. (2025) 17:1525626. doi: 10.3389/fnagi.2025.1525626, 40103932 PMC11913868

[32] Pawluk H Woźniak A Tafelska-Kaczmarek A Kosinska A Pawluk M Sergot K . The role of IL-6 in ischemic stroke. Biomolecules. (2025) 15:470. doi: 10.3390/biom15040470, 40305179 PMC12024898

[33] Kumari S Dhapola R Sharma P Nagar P Medhi B HariKrishnaReddy D. The impact of cytokines in neuroinflammation-mediated stroke. Cytokine Growth Factor Rev. (2024) 78:105–19. doi: 10.1016/j.cytogfr.2024.06.002, 39004599

[34] Zhu H Hu S Li Y Sun Y Xiong X Hu X . Interleukins and ischemic stroke. Front Immunol. (2022) 13:828447. doi: 10.3389/fimmu.2022.828447, 35173738 PMC8841354

[35] Giesers NK Schaeff V Gertz K Endres M Liman TG. Associations of soluble inflammatory and endothelial activation biomarkers with cognitive function over three years after ischemic stroke-PROSCIS-B. Transl Stroke Res. (2025) 16:2249–57. doi: 10.1007/s12975-025-01388-4, 41057606

[36] Narasimhalu K Lee J Leong Y-L Ma L De Silva DA Wong M-C . Inflammatory markers and their association with post stroke cognitive decline. Int J Stroke. (2015) 10:513–8. doi: 10.1111/ijs.12001, 23489773

[37] Loga-Andrijic N Petrovic NT Filipovic-Danic S Marjanovic S Mitrovic V Loga-Zec S. The significance of interleukin-6 and tumor necrosis factor-alpha levels in cognitive impairment among first-ever acute ischaemic stroke patients. Psychiatr Danub. (2021) 33:37–42. 34672270

[38] Irimie C-A Vârciu M Irimie M Ifteni P-I Minea D-I. C-reactive protein and T3: new prognostic factors in acute ischemic stroke. J Stroke Cerebrovasc Dis. (2018) 27:2731–7. doi: 10.1016/j.jstrokecerebrovasdis.2018.05.047, 30056003

[39] Cai Z He W Zhuang F-J Chen Y. The role of high high-sensitivity C-reactive protein levels at admission on poor prognosis after acute ischemic stroke. Int J Neurosci. (2019) 129:423–9. doi: 10.1080/00207454.2018.1538139, 30332913

[40] Zhao P Zhang G Wang Y Wei C Wang Z Zhai W . Peripheral immunity is associated with cognitive impairment after acute minor ischemic stroke and transient ischemic attack. Sci Rep. (2024) 14:16201. doi: 10.1038/s41598-024-67172-w, 39003356 PMC11246473

[41] Xu M Wu Z Wu B Hu Y Duan Q Wang H . Lactate dehydrogenase-to albumin ratio (LAR) is associated with early-onset cognitive impairment after acute ischemic stroke. J Clin Neurosci. (2022) 106:61–5. doi: 10.1016/j.jocn.2022.10.004, 36270094

[42] Zuo L Dong Y Liao X Hu Y Pan Y Yan H . Low HALP (hemoglobin, albumin, lymphocyte, and platelet) score increases the risk of post-stroke cognitive impairment: a multicenter cohort study. Clin Interv Aging. (2024) 19:81–92. doi: 10.2147/CIA.S432885, 38223135 PMC10788070

[43] Xu M Chen L Hu Y Wu J Wu Z Yang S . The HALP (hemoglobin, albumin, lymphocyte, and platelet) score is associated with early-onset post-stroke cognitive impairment. Neurol Sci. (2023) 44:237–45. doi: 10.1007/s10072-022-06414-z, 36192653

[44] GBD 2021 Nervous System Disorders Collaborators. Global, regional, and national burden of disorders affecting the nervous system, 1990-2021: a systematic analysis for the global burden of disease study 2021. Lancet Neurol. (2024) 23:344–81. doi: 10.1016/S1474-4422(24)00038-3, 38493795 PMC10949203

[45] Lohner V Badhwar A Detcheverry FE García CL Gellersen HM Khodakarami Z . Machine learning applications in vascular neuroimaging for the diagnosis and prognosis of cognitive impairment and dementia: a systematic review and meta-analysis. Alzheimer's Res Ther. (2025) 17:183. doi: 10.1186/s13195-025-01815-6, 40775365 PMC12330124

[46] Rost NS Brodtmann A Pase MP van Veluw SJ Biffi A Duering M . Post-stroke cognitive impairment and dementia. Circ Res. (2022) 130:1252–71. doi: 10.1161/CIRCRESAHA.122.319951, 35420911

[47] Bonkhoff AK Grefkes C. Precision medicine in stroke: towards personalized outcome predictions using artificial intelligence. Brain. (2022) 145:457–75. doi: 10.1093/brain/awab439, 34918041 PMC9014757

[48] Folstein MF Folstein SE McHugh PR. “Mini-mental state”. A practical method for grading the cognitive state of patients for the clinician. J Psychiatr Res. (1975) 12:189–98. doi: 10.1016/0022-3956(75)90026-6 1202204

[49] Wei X Liu Y Li J Zhu Y Li W Zhu Y . MoCA and MMSE for the detection of post-stroke cognitive impairment: a comparative diagnostic test accuracy systematic review and meta-analysis. J Neurol. (2025) 272:407. doi: 10.1007/s00415-025-13146-5, 40383729

[50] Dong Y Slavin MJ Chan BP-L Venketasubramanian N Sharma VK Collinson SL . Improving screening for vascular cognitive impairment at three to six months after mild ischemic stroke and transient ischemic attack. Int Psychogeriatr. (2014) 26:787–93. doi: 10.1017/S1041610213002457, 24423626

[51] Clancy U Makin SDJ McHutchison CA Cvoro V Chappell FM Hernández MDCV . Impact of small vessel disease progression on long-term cognitive and functional changes after stroke. Neurology. (2022) 98:e1459–69. doi: 10.1212/WNL.0000000000200005, 35131905 PMC8992602

[52] Zhao X Dai S Zhang R Chen X Zhao M Bergeron MF . Using MemTrax memory test to screen for post-stroke cognitive impairment after ischemic stroke: a cross-sectional study. Front Hum Neurosci. (2023) 17:1195220. doi: 10.3389/fnhum.2023.1195220, 37529406 PMC10387538

[53] Demeyere N Riddoch MJ Slavkova ED Bickerton W-L Humphreys GW. The Oxford cognitive screen (OCS): validation of a stroke-specific short cognitive screening tool. Psychol Assess. (2015) 27:883–94. doi: 10.1037/pas0000082, 25730165

[54] Bisogno AL Franco Novelletto L Zangrossi A De Pellegrin S Facchini S Basile AM . The Oxford cognitive screen (OCS) as an acute predictor of long-term functional outcome in a prospective sample of stroke patients. Cortex. (2023) 166:33–42. doi: 10.1016/j.cortex.2023.04.015, 37295236

[55] Dong Y Ding M Cui M Fang M Gong L Xu Z . Development and validation of a clinical model (DREAM-LDL) for post-stroke cognitive impairment at 6 months. Aging (Albany NY). (2021) 13:21628–41. doi: 10.18632/aging.203507, 34506303 PMC8457606

[56] Wei M Zhu X Yang X Shang J Tong Q Han Q. Development and validation of a novel model to predict post-stroke cognitive impairment within 6 months after acute ischemic stroke. Front Neurol. (2024) 15:1451786. doi: 10.3389/fneur.2024.1451786, 39777316 PMC11703722

[57] Zhang J Kong Z Hong S Zhang Z. Machine learning-based model for prediction of post-stroke cognitive impairment in acute ischemic stroke: a cross-sectional study. Neurol India. (2024) 72:1193–8. doi: 10.4103/ni.ni_987_21, 39690991

