# Supplementary material for: Ischemic stroke-specific cognitive dysfunction: a bibliometric analysis of research evolution
Source: Front Neurol. 2026 Sep 4;17:1891094. doi: 10.3389/fneur.2026.1891094 (PMC13585521; doi:10.3389/fneur.2026.1891094)
Supplement: Supplementary file 1 [file Table_1.docx]

Supplementary Material

# 1 Supplementary Data

## 1.1 Data Cleaning

The raw data downloaded from the WoSCC database may contain duplicates and inconsistent formatting. We merged duplicate entries and corrected irregular expressions. When constructing the country visualization network, we combined “Taiwan” and “Peoples R China” with “China,” and unified “England” and “Scotland” as “UK.” During author analysis, we also merged duplicate names for one author: “Mok, Vincent” and “Mok, Vincent C. T.” Additionally, we changed “Journal of Alzheimers Disease” to “Journal of Alzheimer's Disease.” To refine keywords analysis, terms directly related to the article's focus on cognitive dysfunction after ischemic stroke, such as “stroke,” “ischemic stroke,” “acute stroke,” “ischaemic stroke,” “cognitive dysfunction,” “cognitive impairment,” and “post-stroke cognitive impairment” were excluded. Furthermore, other unrelated terms such as “health,” “disease,” “association,” and “cells.” were omitted to enhance specificity.

# 2 Supplementary Tables

**Supplementary Table 1.** Search strategy and search results from WoSCC database.

| #search | Query | Iterms found |
| --- | --- | --- |
| #3 | #1 AND #2 | 1520 |
| #2 | TS= ("Cognitive Dysfunction" OR "Cognitive Decline" OR "Cognitive Disorder" OR "Cognitive Impairments" OR "post-stroke cognitive impairment" OR "Mental Deterioration" OR "Mild Cognitive Impairment") | 112099 |
| #1 | TS= ("Ischemic Stroke" OR "Acute Ischemic Stroke" OR "Cryptogenic Embolism Stroke" OR "Cryptogenic Ischemic Stroke" OR "Cryptogenic Stroke" OR "Ischaemic Stroke" OR "Wake-up Stroke") | 114458 |

**Supplementary Table 2.** Search strategy and search results from PubMed database.

| #search | Query | Iterms found |
| --- | --- | --- |
| #3 | #1 AND #2 | 1136 |
| #2 | "Cognitive Dysfunction"[Title/Abstract] OR "Cognitive Decline"[Title/Abstract] OR "Cognitive Disorder"[Title/Abstract] OR "Cognitive Impairments"[Title/Abstract] OR "post-stroke cognitive impairment"[Title/Abstract] OR "Mental Deterioration"[Title/Abstract] OR "Mild Cognitive Impairment"[Title/Abstract]) | 109736 |
| #1 | ("Ischemic Stroke"[Title/Abstract] OR "Acute Ischemic Stroke"[Title/Abstract] OR "Cryptogenic Embolism Stroke"[Title/Abstract] OR "Cryptogenic Ischemic Stroke"[Title/Abstract] OR "Cryptogenic Stroke"[Title/Abstract] OR "Ischaemic Stroke"[Title/Abstract] OR "Wake-up Stroke"[Title/Abstract]) | 96502 |

**Supplementary Table 3.** Top 10 most frequent keywords.

| rank | Words | Occurrences | Centrality |
| --- | --- | --- | --- |
| 1 | dementia | 218 | 0.17 |
| 2 | Alzheimer’s disease | 177 | 0.22 |
| 3 | impairment | 150 | 0.10 |
| 4 | risk | 138 | 0.07 |
| 5 | risk factors | 127 | 0.09 |
| 6 | brain | 83 | 0.04 |
| 7 | prevalence | 74 | 0.06 |
| 8 | decline | 73 | 0.04 |
| 9 | small vessel disease | 65 | 0.06 |
| 10 | vascular dementia | 52 | 0.06 |

**Supplementary Table 4.** Top 10 most global cited references.

| Title | Journal | authors | Count | Year | JCR | Impact factor (2024) |
| --- | --- | --- | --- | --- | --- | --- |
| Prevalence, incidence, and factors associated with pre-stroke and post-stroke dementia: a systematic review and meta-analysis | The Lancet Neurology | Pendlebury ST et al. | 1233 | 2009 | Q1 | 45.5 |
| Prevalence and risk factors of cerebral microbleeds: the Rotterdam Scan Study | Neurology | Vernooij MW et al. | 636 | 2008 | Q1 | 8.5 |
| Mediterranean diet, stroke, cognitive impairment, and depression: A meta-analysis | Annals Of Neurology | Psaltopoulou T et al. | 572 | 2013 | Q1 | 7.7 |
| Statin Safety and Associated Adverse Events: A Scientific Statement from the American Heart Association | Arteriosclerosis, Thrombosis, and Vascular Biology | Newman CB et al. | 479 | 2019 | Q1 | 7.4 |
| Stroke Injury,Cognitive Impairment and Vascular Dementia | Biochimica Et Biophysica Acta | Kalaria RN et al. | 362 | 2016 | Q2 | 3.7 |
| Trajectory of Cognitive Decline after Incident Stroke | Journal of the American Medical Association (JAMA) | Levine DA et al. | 332 | 2015 | Q1 | 55 |
| Vascular Cognitive Impairment | Circulation Research | Dichgans M et al. | 326 | 2017 | Q1 | 6.9 |
| Cognitive dysfunction in patients with cerebral microbleeds on t2*-weighted gradient-echo MRI | Brain: a Journal of Neurology | Werring DJ et al. | 324 | 2004 | Q1 | 11.7 |
| Post-Stroke Cognitive Impairment and Dementia | Circulation Research | Rost NS et al. | 320 | 2022 | Q1 | 16.2 |
| Epidemiology of stroke in Europe and trends for the 21st century | La Presse MÉDicale | Béjot Y et al. | 314 | 2016 | Q1 | 3.4 |

**Supplementary Table 5.** Top 10 most frequently co-cited references.

| Title | Journal | authors | Count | Centrality | Year | JCR | Impact factor (2024) |
| --- | --- | --- | --- | --- | --- | --- | --- |
| Post-stroke dementia-a comprehensive review | BMC Medicine | Mijajlović MD et al. | 40 | 0.30 | 2017 | Q1 | 8.3 |
| Incidence and prevalence of dementia associated with transient ischaemic attack and stroke: analysis of the population-based Oxford Vascular Study | The Lancet Neurology | Pendlebury ST et al. | 39 | 0.13 | 2019 | Q1 | 45.5 |
| Post-Stroke Cognitive Impairment and Dementia | Circulation Research | Rost NS et al. | 34 | 0.02 | 2022 | Q1 | 16.2 |
| Profile of and risk factors for poststroke cognitive impairment in diverse ethnoregional groups | Neurology | Jessica W Lo et al. | 29 | 0.03 | 2019 | Q1 | 8.5 |
| Strategic infact location for post-stroke cognitive impairment: a pooled analysis of individual patient data from 12 acute ischaemic stroke cohorts | The Lancet Neurology | Nick A Weaver et al. | 25 | 0.08 | 2021 | Q1 | 45.5 |
| Global, reginal, and national burden of stroke and its risk factors, 1990-2019: a systematic analysis for the Global Burden of Disease Study 2019 | The Lancet Neurology | Feigin VL et al. | 21 | 0.01 | 2021 | Q1 | 45.5 |
| Prevalence, incidence, and factors associated with pre-stroke and post-stroke dementia: a systematic review and meta-analysis | The Lancet Neurology | Pendlebury ST et al. | 19 | 0.28 | 2009 | Q1 | 45.5 |
| Post-Stroke Cognitive Impairment: A Review Focusing on Molecular Biomarkers | Journal of Molecular Neuroscience | Zhang XX et al. | 17 | 0.04 | 2020 | Q2 | 2.7 |
| Trajectory of Cognitive Decline After Incident Stroke | Jama-Journal of the American Medical Association | Levine DA et al. | 16 | 0.02 | 2015 | Q1 | 55.0 |
| Systematic review and meta-analysis of the prevalence of cognitive impairment no dementia in the first year post-stroke | European Stroke Journal | Sexton E et al. | 16 | 0.03 | 2019 | Q1 | 4.5 |
